# Glutathionylation of the L-type Ca^2+^ Channel in Oxidative Stress-Induced Pathology of the Heart

**DOI:** 10.3390/ijms151019203

**Published:** 2014-10-22

**Authors:** Victoria P. A. Johnstone, Livia C. Hool

**Affiliations:** 1 School of Anatomy, Physiology and Human Biology, the University of Western Australia, Crawley 6009, WA, Australia; E-Mail: vicky.johnstone@uwa.edu.au; 2 Victor Chang Cardiac Research Institute, Darlinghurst 2010, NSW, Australia

**Keywords:** oxidative stress, L-type calcium channel, reactive oxygen species (ROS), glutathionylation, calcium, ROS-induced ROS release

## Abstract

There is mounting evidence to suggest that protein glutathionylation is a key process contributing to the development of pathology. Glutathionylation occurs as a result of posttranslational modification of a protein and involves the addition of a glutathione moiety at cysteine residues. Such modification can occur on a number of proteins, and exerts a variety of functional consequences. The L-type Ca^2+^ channel has been identified as a glutathionylation target that participates in the development of cardiac pathology. Ca^2+^ influx via the L-type Ca^2+^ channel increases production of mitochondrial reactive oxygen species (ROS) in cardiomyocytes during periods of oxidative stress. This induces a persistent increase in channel open probability, and the resulting constitutive increase in Ca^2+^ influx amplifies the cross-talk between the mitochondria and the channel. Novel strategies utilising targeted peptide delivery to uncouple mitochondrial ROS and Ca^2+^ flux via the L-type Ca^2+^ channel following ischemia-reperfusion have delivered promising results, and have proven capable of restoring appropriate mitochondrial function in myocytes and *in vivo*.

## 1. Introduction

Oxidative stress is a key feature underlying many different forms of cardiac pathology and represents an imbalance between the production of reactive oxygen species (ROS) and the cell’s inherent antioxidant defense system [1]. Reactive oxygen species are chemically reactive molecules that are derived from the reduction of molecular oxygen [2]. Under normal conditions the cellular concentrations of ROS are maintained within a narrow range [3] and act as critical mediators of various processes, including cell proliferation, differentiation and apoptosis, gene expression and cell metabolism [4,5,6,7,8]. Increases in intracellular ROS during pathological states lead to alterations in the regulation of signaling pathways, coupled with redox-modification of critical cellular proteins [9]. Much attention has been paid to the L-type Ca^2+^ channel as a target, largely because it is the primary means of Ca^2+^ entry into cardiomyocytes. Calcium is vital for cardiac excitation-contraction coupling, but is also a key regulator of mitochondrial ROS production. The interplay between ROS and Ca^2+^ in cardiomyocytes is therefore a critical determinant of cardiac function [10,11]. In this review article, the cross-talk between ROS and Ca^2+^ in mediating cardiac pathology will be examined, and particular emphasis will be placed on ROS-dependent changes in L-type Ca^2+^ channel function including glutathionylation. Finally, the use of novel interventions to alleviate the effects of oxidative stress-induced changes in L-type calcium channel function will be discussed.

## 2. The Role of Calcium and Reactive Oxygen Species (ROS) in Oxidative Stress Responses

### 2.1. Calcium Homeostasis and Myocyte Contraction

Calcium is a critical regulator of cardiac excitation-contraction coupling. In ventricular myocytes, depolarisation during the rapid rising phase of the action potential induces inward flux of calcium via the L-type Ca^2+^ channel, and this leads to further downstream calcium release via ryanodine receptors on the sarcoplasmic reticulum [12]. This calcium-induced calcium release (CICR) results from a close association between the L-type Ca^2+^ channel and ryanodine receptors, and provides rapid amplification of the initial calcium trigger event, such that activation of a single channel may initiate release from an estimated 6–20 ryanodine receptors [12,13]. The rise in intracellular calcium activates contraction by the binding of calcium to troponin, a component of the thin filament complex. Muscle fibers are formed from overlapping strands of contractile proteins comprising a thick filament composed of myosin and a thin filament made of actin and troponin/tropomyosin. Calcium binding to troponin induces an allosteric modification of the troponin/tropomyosin complex. This unblocks the myosin binding sites on the actin filament, and myosin is then powered by hydrolysing adenosine triphosphate (ATP) to move along these binding sites, resulting in muscle contraction [13]. Contraction is closely followed by relaxation and removal of free cytosolic calcium. This is predominantly mediated by the sarcoplasmic reticulum Ca^2+^-ATPase, with additional extrusion via the Na^+^/Ca^2+^ exchanger, the sarcolemmal Ca^2+^-ATPase (and possibly the mitochondrial Ca^2+^ uniporter [14]) playing a comparatively minor role [12,15].

### 2.2. Ca^2+^ and ROS-Induced ROS-Release

Aside from its role in contraction, calcium is also critical for the production of ATP and ROS. Mitochondrial calcium entry via the mitochondrial Ca^2+^ uniporter (MCU) stimulates the tricarboxylic acid (TCA) cycle and oxidative phosphorylation [16,17,18,19], and is specifically required for activation of three key dehydrogenases of the TCA cycle, pyruvate dehydrogenase, isocitrate dehydrogenase and oxoglutarate dehydrogenase [19,20], as well as the ATP synthase (Complex V of the electron transport chain) [18] and the adenine nucleotide translocase [16]. Activation of the TCA cycle causes increased production of NADH, which triggers movement of electrons down Complexes I–IV of the electron transport chain. Electrons are also fed in to the electron transport chain via Complex II as a result of the conversion of succinate to fumarate within the TCA cycle. Complex IV is the terminal electron acceptor, which acts to convert oxygen to water. During this process Complexes I, III and IV pump protons into the intermembrane space from the mitochondrial matrix, resulting in an increased proton motive force that exists as an electrochemical potential, also known as mitochondrial membrane potential [21]. This potential difference allows for the production of ATP from ADP at Complex V [21,22,23]. During this process of electron transfer, electrons leak from the respiratory chain and react with molecular oxygen to form superoxide [9,11,24,25,26]. Superoxide is rapidly converted to more stable H_2_O_2_ by manganese superoxide dismutase, which is present at a high concentration within the mitochondrial matrix [9,24,27,28]. Hydrogen peroxide then readily diffuses into the cytoplasm, where it is either partially reduced to the hydroxyl radical or fully catabolised to water via activity of the enzyme catalase [2]. Reactive hydroxide and H_2_O_2_ are then free to modify the function of critical cellular proteins [29].

**Figure 1:**
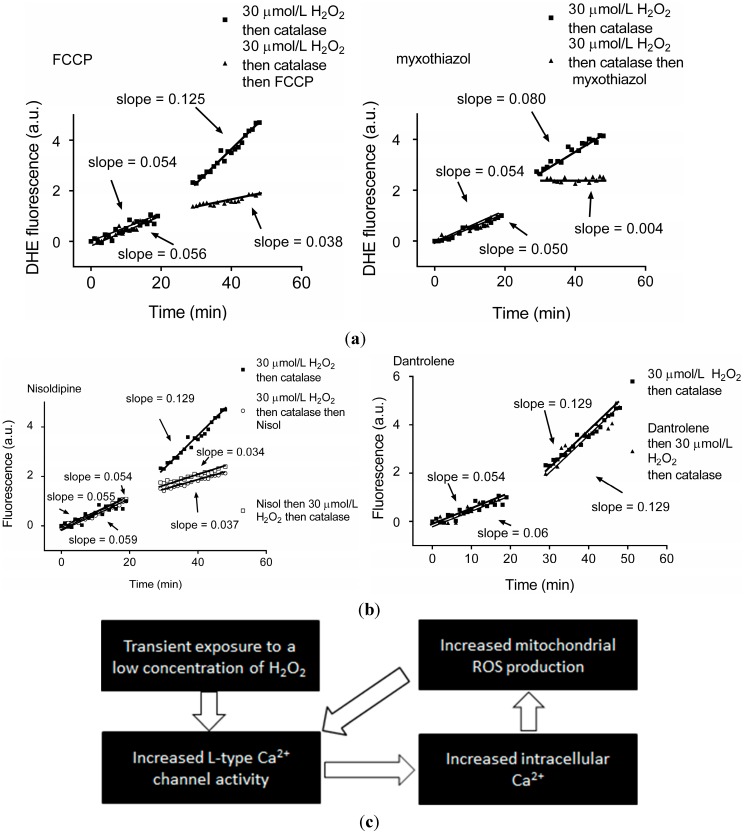
Transient exposure to H_2_O_2_ leads to L-type Ca^2+^ channel-dependent increase in mitochondrial superoxide, triggering ROS-induced-ROS release. (**a**) dihydroethidium (DHE) fluorescence recorded from individual guinea pig cardiomyocytes before and after exposure to 30 µM H_2_O_2_ for 5 min followed by 10 U/mL catalase for 5 min then 2 nM FCCP (an uncoupler of oxidative phosphorylation) as indicated (**left panel**) or 7 nM myxothiazol (to block electron transport at mitochondrial complex III) (**right panel**); (**b**) DHE fluorescence recorded from individual guinea pig cardiomyocytes before and after exposure to 30 µM H_2_O_2_ for 5 min followed by 10 U/mL catalase for 5 min and the L-type Ca^2+^ channel antagonist nisoldipine (nisol; 2 µM) as indicated (**left panel**) or ryanodine receptor antagonist dantrolene (20 µM; **right panel**); and (**c**) Schematic illustrating the persistent increase in intracellular ROS and intracellular Ca^2+^ following a transient H_2_O_2_ exposure or ROS-induced ROS-release. Reproduced with permission from [40].

In pathological states, elevated levels of ROS can be amplified via a process known as ROS-induced ROS release (RIRR) [30,31,32]. The mechanisms underlying RIRR are commonly attributed to a positive feedback loop, in which the mitochondria are both the producer and the target of ROS [32,33,34]. However there is good evidence to suggest that an additional pathway of RIRR is via cross-talk between mitochondrial ROS and the L-type Ca^2+^ channel. The L-type Ca^2+^ channel contains a number of cysteines that are putative targets for modification under oxidising conditions, and acute exposure to H_2_O_2_ or thiol-oxidising compounds has been shown to increase L-type Ca^2+^ channel current [35,36,37,38]. Conversely, channel activity is decreased following exposure of the pore-forming α_1C_ subunit of the human L-type Ca^2+^ channel expressed in HEK293 cells to a hypoxic insult [39]. Acute hypoxia decreases basal L-type Ca^2+^ channel current in isolated guinea pig ventricular myocytes without altering the current-voltage relationship [35]. Therefore there is good evidence that the channel is responsive to changes in the cell’s redox state. In order to further elucidate the interaction between the L-type Ca^2+^ channel and mitochondrial ROS production, we investigated the effect of a transient exposure of guinea pig cardiac myocytes to a concentration of H_2_O_2_ that was insufficient to induce apoptosis or necrosis, but mimicked an ischemia/reperfusion (*I*/*R*) injury, in which return of blood flow to the ischemic heart during reperfusion induces a burst of ROS [40]. Myocytes were exposed to 30 µM H_2_O_2_ for 5 min, followed by 10 U/mL catalase for 5 min to degrade the H_2_O_2_. Transient exposure of the myocytes to H_2_O_2_ significantly increased cellular superoxide levels, assessed by changes in dihydroethidium (DHE) fluorescence. The increase was attenuated when the myocytes were exposed to the electron transport chain uncoupler, carbonyl cyanide *p*-(trifluoromethoxy) phenyl-hydrazone (FCCP), or the mitochondrial inhibitor myxothiazol (Figure 1a) [40], identifying a mitochondrial origin for the increased superoxide consistent with the RIRR hypothesis. In addition, inhibition of mitochondrial calcium uptake using Ru360 (to block the MCU) resulted in an attenuation of the superoxide signal [40]. Consistent with this, acute treatment of myocytes with H_2_O_2_ induced an increase in free cytosolic calcium, assessed using the fluorescent free calcium indicator Fura-2 [40]. The calcium source required for the increase in cellular superoxide was subsequently identified as the L-type Ca^2+^ channel, as the increase in superoxide following transient exposure to H_2_O_2_ was abolished when the L-type Ca^2+^ channel was blocked with the channel antagonist nisoldipine, but not when calcium released from the sarcoplasmic reticulum via the ryanodine receptor was blocked with dantrolene (Figure 1b) [40]. A direct connection between the oxidative stress and alteration in L-type Ca^2+^ channel activity was subsequently established using whole-cell patch clamp recordings. Acute exposure of the myocytes to H_2_O_2_ increased basal activity of the L-type Ca^2+^ channel [29]. The increase in basal current density persisted for at least nine hours post-exposure due to the increase in mitochondrial superoxide, and was reversible.

These findings provide direct evidence that ROS generated by the mitochondria can act as potent and persistent regulators of L-type Ca^2+^ channel function (Figure 1c). It is worth noting that similar associations between ROS and voltage-gated calcium channels have been reported elsewhere. Neuronal P/Q-type Ca^2+^ channels expressed in *Xenopus* oocytes exhibit enhanced whole-cell currents following exposure to extracellular H_2_O_2_, as a result of an increase in channel open probability [41]. In other cell types there appears to be a reverse association between calcium and ROS under certain conditions. Exposure of L-type Ca^2+^ channels expressed in Chinese Hamster Ovary (CHO) cells to sulphydryl-oxidising agents inhibits channel activity as assessed using whole-cell and single channel recording techniques [42].

Cross-talk between calcium and ROS is therefore likely to be a mechanism that is highly conserved between systems, to allow for fine-tuning of cell redox state and calcium homeostasis.

### 2.3. Post-Ischemic Persistent Elevation of ROS and Ca^2+^ Is Pathological

**Figure 2:**
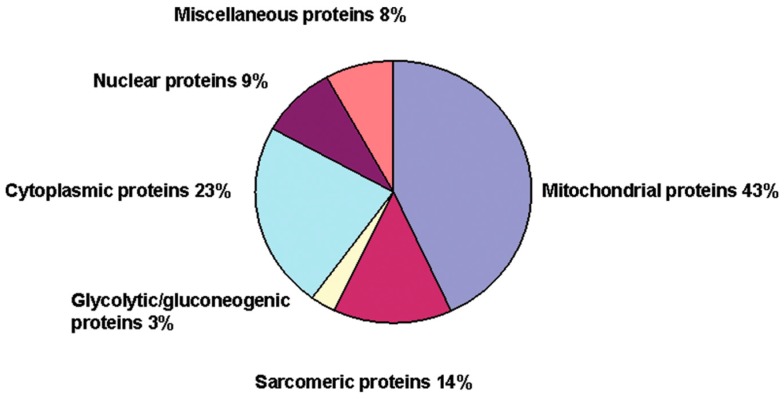
Transient exposure of myocytes to H_2_O_2_ alters mitochondrial protein synthesis. Exposure of guinea-pig myocytes to 30 µM H_2_O_2_ for 5 min followed by 10 U/mL catalase for 5 min is sufficient to induce alterations in cellular protein synthesis consistent with the development of myocyte hypertrophy. iTRAQ-facilitated proteomics analysis indicated that the majority of changes in protein synthesis are mitochondrial in origin. For further details see text. Reproduced with permission from [50].

Cardiac hypertrophy is a common consequence of physiological increases in demand and/or pathological insults to the heart, and is characterised by an increase in the size of individual cardiac myocytes (without a change in myocyte number), enhanced protein synthesis and disorganisation of the sarcomere [43,44]. Many hypertrophic signaling pathways are triggered by an increase in intracellular calcium concentration and/or an elevation in ROS [45,46,47], and contribute to the development of ischemic heart disease and congestive heart failure [1,48,49]. Given the link between elevated calcium, ROS and cardiac dysfunction, we subsequently confirmed that the transient exposure to H_2_O_2_ and associated increase in superoxide [40] was sufficient to induce pathology at 48 h post-insult. Transient exposure of guinea pig myocytes to 30 µM H_2_O_2_ for 5 min resulted in a small but significant increase in the size of individual cardiomyocytes when imaged 48 h post-exposure [50]. Transient H_2_O_2_ exposure was also associated with an approximate two-fold increase in protein synthesis (assessed as incorporation of [^3^H]leucine). The increase was attenuated following L-type Ca^2+^ channel inhibition with nisoldipine but was unaffected by inhibition of the ryanodine receptor using dantrolene, indicating that the source of calcium was via the L-type Ca^2+^-channel and not intracellular Ca^2+^ stores [50]. Inhibition of downstream Ca^2+^-dependent signaling pathways using the Ca^2+^/calmodulin-dependent protein kinase II (CAMKII) inhibitor KN-62 also suppressed the enhancement of protein synthesis, consistent with previous reports identifying CAMKII signaling as a core mechanism underlying the development of cardiac hypertrophy and heart failure [51]. In order to identify and quantitate the expression levels of proteins 48 h after myocyte treatment with H_2_O_2_, a combined proteomics/isobaric tag for relative and absolute quantification (iTRAQ) based screening method was utilised. Using this approach, a total of 35 proteins were identified with altered expression, the majority of which were mitochondrial in origin (Figure 2) [50]. The proteins identified were predominantly involved in cellular metabolism, and included TCA cycle enzymes and oxidative phosphorylation proteins. Although there was no microscopic evidence of actin disorganisation at 48 h post-H_2_O_2_ exposure, iTRAQ analysis revealed alterations in the expression of sarcomeric proteins, including β-myosin heavy chain and troponin C, both of which are involved in sarcomeric disarray in advanced stages of cardiac hypertrophy [52]. The protein expression profile at 48 h after exposure to H_2_O_2_ offers a snapshot of the dynamic acute phase of induction of hypertrophy, and more pronounced increases in cell size and visible disorganisation of the sarcomere would likely become apparent at later time points. Therefore H_2_O_2_-induced persistent activation of the L-type Ca^2+^ channel is indeed sufficient to induce cardiac hypertrophy, and cross-talk between the L-type Ca^2+^ channel and mitochondrial ROS is critical in the progression of cardiac pathology.

### 2.4. Mitochondrial Complex III Is the Locus for Superoxide Generation

Under normal physiological conditions, leak of electrons from the electron transport chain occurs at mitochondrial Complexes I and III [2,53,54]. These electrons then react with molecular oxygen to form superoxide. To ascertain the site of increased superoxide production following transient H_2_O_2_ exposure, strategic pharmacological inhibition of the individual mitochondrial complexes was used in combination with fluorescent detection of changes in DHE signal to assess alterations in superoxide production. We had previously demonstrated that nicotinamide adenine dinucleotide phosphate NADPH-oxidase was not the source of superoxide [40]. Therefore we used diphenyleneiodonium chloride (DPI) to block electron flow prior to the superoxide generation site at Complex I, and this effectively attenuated the increase in DHE signal in response to transient H_2_O_2_ exposure [55]. Block of electron flow after the superoxide generation site of Complex I using rotenone also attenuated the H_2_O_2_-induced increase in superoxide signal [55]. Therefore the locus of increased superoxide was concluded to be either at or beyond Complex I. To examine whether Complex III was the site of enhanced superoxide production, myocytes were exposed to 7 nM myxothiazol and stigmatellin (a concentration that did not alter mitochondrial membrane potential), to block electron flow at the Q_0_ superoxide site of Complex III. Both compounds successfully attenuated the increase in superoxide [55]. Blocking of electron flow at the Q*_i_* superoxide generation site of Complex III (which is downstream from Q_0_) using antimycin A did not alter the increase in superoxide production, however, nor did application of sodium cyanide to block Complex IV [55]. This identified the Q_0_ superoxide generation site of Complex III as the locus of increased superoxide production, since blocking of electron flow beyond this site had no effect on the production of superoxide.

In order to provide unequivocal confirmation of the Q_0_ site of Complex III as the predominant source of superoxide, simultaneous whole-cell patch clamp and fluorescence imaging experiments were performed in which the Q_0_ site was pharmacologically isolated. To achieve this, cardiac myocytes were perfused with the Complex II substrate succinate intracellularly via the patch pipette, while the extracellular medium was supplemented with the Complex I inhibitor rotenone and the Complex III Q*_i_* site inhibitor antimycin A. This approach allowed for block of electron flow from Complex I and from Complex III Q*_i_* site onwards, leaving the Q_0_ site of complex III intact. Under these conditions, transient exposure to H_2_O_2_ resulted in a similar increase in superoxide signal as had previously been recorded from cells that were not patch-clamped. The increase in superoxide was blocked when the myocytes were exposed to extracellular myxothiazol (to block electron flow at Q_0_) prior to H_2_O_2_ insult.

In order to develop appropriate therapies to effectively limit the development of ROS-induced cardiac hypertrophy, it is necessary to first understand the progression of events involved during the acute phase of oxidative stress. Identification of the Q_0_ site of Complex III as the locus for increased superoxide production is an important step towards the development of therapeutic strategies for ROS-induced hypertrophy, which have otherwise proven elusive. To that end, there is limited evidence to suggest that myxothiazol is capable of decreasing mitochondrial ROS production during an ischemic insult in an experimental setting [56]. However targeting the electron transport chain may yet prove to be a viable strategy.

## 3. Glutathionylation of the L-type Ca^2+^ Channel during Oxidative Stress

### 3.1. Overview of Protein Glutathionylation

Hydrogen peroxide is responsible for mediating many cellular redox reactions, and acts as an efficient second messenger that provides rapid communication within and between cells [57]. One of the most common outcomes of a rise in cellular oxidant levels is the modification of redox-sensitive proteins via a process known as *S*-glutathionylation [58]. Glutathionylation occurs as a posttranslational modification of proteins at the cysteine residues by adding a glutathione (GSH, γ-glutamylcysteinylglycine) moiety [58,59,60]. Glutathione is a ubiquitous tripeptide that acts as an inherent antioxidant, and works in conjunction with oxidised glutathione (glutathione disulphide, GSSG) as an intracellular redox buffer [58,61]. The ratio of GSH to GSSG therefore contributes to the redox potential of the cell, and during periods of oxidative stress, ROS oxidises GSH to GSSG (causing a decrease in the GSH/GSSG ratio) via Equation (1) [62].

(1) $$2GSH+H_{2}O_{2}↔GSSG+2\;H_{2}O$$

A critical role of the GSH-GSSG redox couple is to facilitate reversible *S*-glutathionylation of proteins (Pr) [60]. One means by which glutathionylated proteins (PrSSG) are formed is via direct reaction of a cysteine residue on a protein (PrSH) with GSSG via a thiol-disulfide exchange reaction. (Equation (2)) [61,63,64]. Alternatively, proteins can be glutathionylated via interaction between oxidised protein thiols and GSH [61].

(2) PrSH + GSSG ↔ PrSSG + GSH

During periods of oxidative stress, ROS oxidises GSH to GSSG (resulting in a decrease in the ratio of GSH to GSSG), and leading to increased glutathionylation of proteins [62]. It is important to note however that the kinetics of protein glutathionylation are influenced by other factors beyond the redox state of the cell, including the p*K*_a_ of the sulfhydryl (–SH) group of protein cysteine residues and the steric location of cysteine residues within the protein [61]. These factors in conjunction with the cell redox potential dictate the probability of protein glutathionylation. To date, a number of proteins have been identified that undergo *S*-glutathionylation, often in response to oxidative stress. *S*-Glutathionylation of proteins can induce a range of consequences [59,60,65], including a downregulation [66,67] or upregulation [68] of enzymatic activity, altered DNA binding by transcription factors [69,70] and increased [71] or decreased [72] protein stability. Whilst there are many cellular modifications that occur as a result of oxidative stress, available evidence suggests that glutathionylation may predominate as a critical signaling mechanism in cardiac disease [73].

### 3.2. Glutathionylation of the L-type Ca^2+^ Channel

Given the strong evidence that protein glutathionylation is a key process underlying oxidative stress-induced disease, we hypothesised that the H_2_O_2_-induced persistent increase in L-type Ca^2+^ channel activity [40] was due to glutathionylation of the channel. To assess this, we first examined whether the purified channel protein was a candidate for glutathionylation *in vitro*. The channel was isolated from guinea pig heart homogenates and exposed to 1 mM GSH and 30 µM H_2_O_2_. Immunoblot analysis revealed that the channel was significantly glutathionylated following this exposure [74].

**Figure 3:**
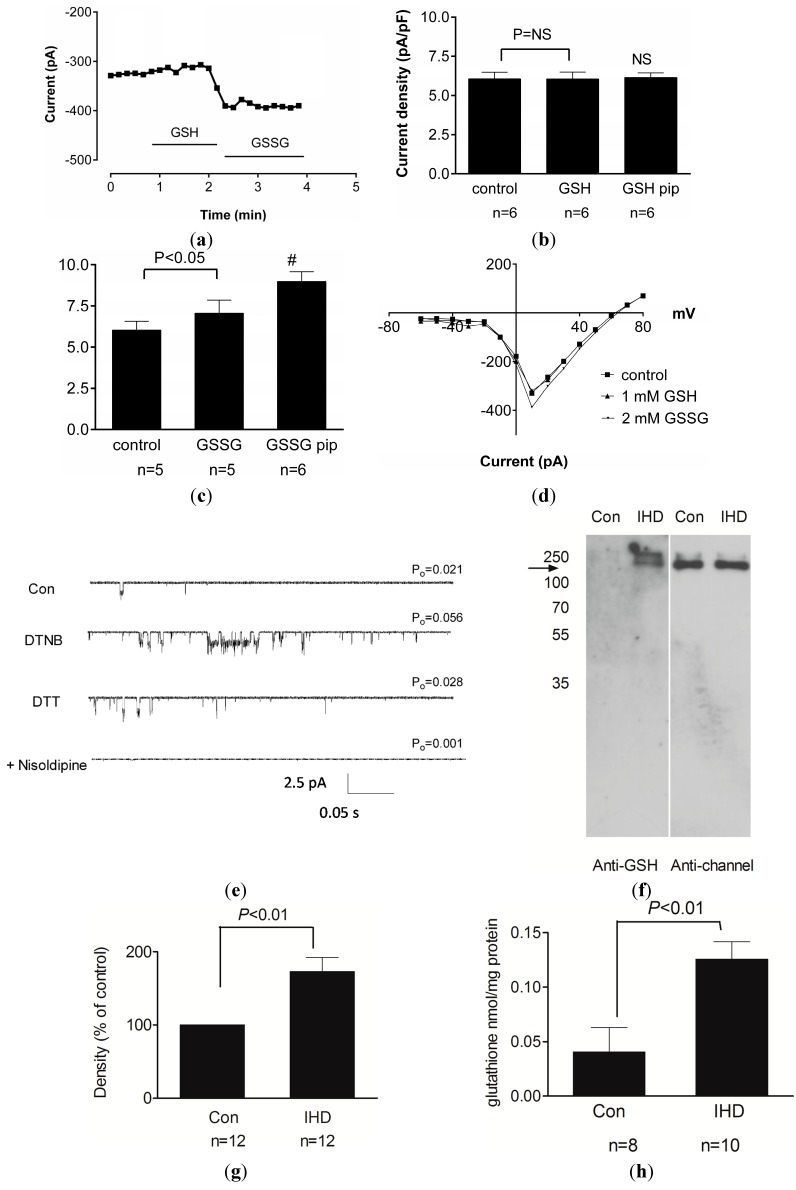
Oxidative stress causes persistent enhancement of the L-type Ca^2+^ channel via ROS-mediated glutathionylation. (**a**) Time course of changes in membrane current recorded from guinea pig cardiac myocytes during extracellular exposure to 1 mM GSH followed by 2 mM GSSG as indicated; (**b**) Mean (±SEM) of L-type Ca^2+^ channel current density under control conditions (no drugs) and after exposure to GSH applied either extracellularly or in the patch pipette (GSH pip). NS, not significant; (**c**) Mean (±SEM) of L-type Ca^2+^ channel current density under control conditions (no drugs) and after exposure to GSSG applied either extracellularly or in the patch pipette (GSSG pip). #, *p* < 0.05 *vs.* control; (**d**) Current-voltage relationship for representative myocytes during voltage steps from −60 to +80 mV and exposure to GSH or GSSG as indicated; (**e**) Representative single channel currents recorded at −100 mV in the absence and presence of 200 µM DTNB followed by 1 mM DTT and then 2 µM nisoldipine. The open probability (*P*_0_) for each treatment is indicated; (**f**) Immunoblot demonstrating glutathionylation of protein after probing with anti-GSH antibody (**left**) and anti-channel antibody (**right**) in immunoprecipitated Ca_v_1.2 channel protein samples from control nonischemic human heart (Con) and ischemic human heart (IHD); (**g**) Densitometry analysis of immunoblots for glutathionylated protein band normalised to the channel protein in the same lane for Con samples and IHD samples (mean ± SEM); and (**h**) Concentration of protein-glutathione mixed-disulfides in channel protein from Con and IHD heart samples (mean ± SEM). Reproduced with permission from [74].

We next examined the effects of GSH and GSSG on the function of the native channel using the patch clamp technique *in vitro*. Exposure of ventricular myocytes to 1 mM GSH in the extracellular solution resulted in a small decrease in basal channel activity, with no change in activity when the GSH was applied intracellularly via the patch pipette (Figure 3a,b) [74]. External application of GSSG produced a significant increase in the peak inward current when the cell voltage was stepped to +10 mV, with no change in the current-voltage relationship of the channel (Figure 3a,c,d) [74]. This effect was amplified when GSSG was applied intracellularly via the patch pipette, suggesting that the cytoplasmic portion of the L-type Ca^2+^ channel is more sensitive to redox modification (Figure 3c). The increase in current observed with GSSG application is consistent with prior reports by us, and others, that H_2_O_2_ exposure induces a persistent enhancement of channel function [38,40,75,76]. We subsequently confirmed that GSSG application was sufficient to increase intracellular calcium, assessed as changes in Fura-2 fluorescence, consistent with a ROS-induced potentiation of L-type Ca^2+^ channel activity. This increase in intracellular calcium was attenuated with glutaredoxin, an agent that induces reduction of GSSG to GSH [77]. In contrast GSH application had no effect on intracellular calcium [74]. Therefore glutathionylation of the L-type Ca^2+^ channel alters macroscopic channel function and intracellular calcium during oxidative stress.

### 3.3. Functional and Clinical Consequences of L-type Ca^2+^ Channel Glutathionylation

To verify that the effects of GSSG and H_2_O_2_ were due to a direct effect on the channel protein, we reconstituted the purified long *N*-terminal (NT) isoform of the human Ca_v_1.2 (α_1C_ subunit) into proteoliposomes [74]. This strategy allowed for functional analysis of channel currents to be performed in the absence of other cellular proteins, such as the auxiliary subunits that can modulate biophysical channel properties and are reported to be vulnerable to glutathionylation [78]. Exposure of the channel to either GSH, nisoldipine, or the thiol reducing agent dithiothreitol (DTT) significantly decreased channel open probability, whilst GSSG, H_2_O_2_ and the thiol oxidising agent 5,5'-dithio-bis(2-nitrobenzoic acid) (DTNB) induced an increase in channel open probability (Figure 3e). These changes occurred in the absence of any change in the current-voltage sensitivity of the channel.

We subsequently confirmed that an experimental ischemia reperfusion (*I*/*R*) protocol was sufficient to induce glutathionylation of the L-type Ca^2+^ channel [74]. In these experiments, guinea pig hearts were retrogradely perfused via the aorta on a Langendorff apparatus and exposed to 30 min of no-flow ischemia followed by 30 min of reperfusion. Elevated levels of creatine kinase (CK) and lactate dehydrogenase (LDH) were measured in the coronary effluent, indicative of myocardial damage [79]. Further analysis revealed an increase in the level of glutathionylation of the L-type Ca^2+^ channel following *I*/*R* injury, and a glutathione recycling assay was used to confirm a significant increase in the level of protein-glutathione mixed disulfides [74]. The modifications were also confirmed to occur in human hearts following oxidative stress. A significant increase in the level of glutathionylation of the L-type Ca^2+^ channel was found in ventricular samples from ischemic human hearts when compared to non-ischemic controls (Figure 3f–h) [74]. This finding provides confirmation that the persistent redox-dependent alterations in channel function we described in the laboratory setting are clinically relevant.

A number of proteins have been demonstrated to undergo reversible glutathionylation. These proteins often tend to cluster into several categories, and are frequently involved in energy metabolism, cell signaling, cytoskeletal function, protein folding and editing, redox balance and ion channel modification [61,80]. Thus far we have limited knowledge of how structure and function of these proteins are altered by glutathionylation. In the case of ion channels, there is good evidence to suggest that glutathionylation can inhibit ion flux via an alteration in channel conductance. For example, the vascular ATP-sensitive potassium channel (K_ATP_) is glutathionylated at Cys176, which is located in the critical region of the inner helix, close to both the activation and hinge gate residues [81]. *In silico* modelling of the channel has demonstrated that binding of the GSH moiety to Cys176 prevents the channel from entering its open state and retains it in a closed conformation. Other channels exhibit enhanced activity after glutathionylation, and structural changes that affect channel kinetics are likely to underlie the enhancement. For example, the neuronal Kv4 voltage-gated potassium channel contains an auxiliary subunit (dipeptidyl peptidase-like protein 6; DPP6a) which is glutathionylated at Cys13, resulting in a slowing of inactivation kinetics and increase in peak current amplitude [78]. These effects can be fully reversed using the thiol reducing agent DTT [78].

The α_1C_ subunit of the L-type Ca^2+^ channel contains 48 cysteines, approximately 38 of which are located at sites that could be accessible for glutathionylation. It is likely that glutathionylation at one or more of these sites therefore induces a structural change that results in an increase in the open probability of the channel, without altering the voltage sensitivity. This would allow for a greater amount of calcium influx into the cell, without altering single channel conductance. The L-type Ca^2+^ channel therefore represents a viable clinical target following ischemia reperfusion injury, and tailoring therapies to reduce or inhibit redox-dependent modifications of the channel may prevent the activation of signaling pathways and development of hypertrophy.

### 3.4. S-Nitrosylation of the L-type Ca^2+^ Channel as an Alternate Means of Channel Modification during Oxidative Stress

Nitric oxide (NO) signaling is important for a diverse range of physiological processes [82]. Signaling is mediated by a range of molecules, including *S*-nitrosothiols which are derivatives of the free radical NO [83]. *S*-Nitrosothiols serve as donors of the nitrosonium ion (NO^+^), and can facilitate *S*-nitrosylation of proteins via the addition of a nitroso group to a sulfur atom of an amino acid. This process is reversible and results in post-translational modification of the target protein. There is some evidence to suggest that *S*-nitrosylation of the L-type Ca^2+^ channel can occur following changes in oxygen tension, and that this leads to an inhibition of channel current [84,85]. Contrary to these reports, we find no evidence for a role for NO in the regulation of basal L-type Ca^2+^ currents or the inhibition of current induced by hypoxia [35]. In addition we report no effect of NO inhibitors on the L-type Ca^2+^ current in the presence of acute exposure to H_2_O_2_ [40]. It is possible that variation in the extent of oxidative stress and in the experimental preparation used may account for these differences.

## 4. Novel Interventions to Alleviate Ischemic Injury

### 4.1. Overview of Ischemia-Reperfusion Injury

Ischemic heart disease is characterised by reduced coronary blood flow and is a leading cause of mortality and morbidity worldwide [43,86]. Ischemic injury is associated with atherosclerosis, but is also an unfortunate consequence of some clinical procedures (e.g., angioplasty, cardiopulmonary bypass). In such circumstances timely reperfusion of the heart is essential to reduce further cardiac damage, however reflow into the ischemic heart paradoxically leads to additional myocardial injury [87,88]. Cardiac tissue has been characterised histologically following reperfusion injury and is typified by unique features, including contraction bands in the contractile proteins, calcific granules within mitochondria and the disruption of the sarcoplasmic and mitochondrial membranes [89,90,91,92,93]. Functional changes to cardiac tissue following reperfusion include depressed contractility, reduced coronary flow and altered vascular reactivity [94,95,96]. A number of mechanisms have been proposed as contributors to *I*/*R* injury, including activation of inflammatory cascades [88], calcium overload [97], the no reflow phenomenon [98,99] and endothelial dysfunction [100,101], but there is good evidence to suggest that the increased production of ROS is a primary factor [102]. A critical study by Bolli and colleagues [103] provided unequivocal confirmation that large quantities of ROS are produced in the first few minutes following reperfusion, and that this is an important determinant of reperfusion injury. Increased ROS production following reperfusion alters the redox state of the cell, and induces a number of cellular effects including an upregulation of protein glutathionylation and impaired calcium homeostasis.

### 4.2. Reduced Ischemia-Reperfusion Injury Using Targeted Peptide Delivery

Our experimental work has identified that persistent activation of the L-type Ca^2+^ channel via redox-dependent glutathionylation is critical in triggering downstream cascades that lead to myocyte dysfunction [74]. Features of this dysfunction include impaired calcium homeostasis and mitochondrial function, and increased protein synthesis and cell size, consistent with the development of myocyte hypertrophy [40,50,74,104,105]. We have demonstrated that some of these features can be attenuated using targeted peptide delivery to prevent increased activation of the L-type Ca^2+^ channel during periods of oxidative stress.

The cardiac L-type Ca^2+^ channel is a heterotetramer comprised of the α_1C_, α_2_δ and β_2_ subunits. The α_1C_ subunit contains the pore-forming and voltage-sensing regions responsible for ion conductance, and contains the binding sites for channel modulating drugs and toxins [15,106,107]. The α_2_δ subunit regulates channel turnover at the membrane and is required for appropriate channel trafficking [108]. The β_2_ subunit binds to the α_1_ subunit at the alpha-interacting domain (AID) and assists with the trafficking and insertion of the α_1C_ subunit into the membrane [109]. The β_2_ subunit also regulates channel inactivation, and couples the channel to cytoskeletal proteins [110]. There is good evidence to suggest that the β_2_ subunit of the channel interacts with F-actin via a 700 kDa stabilizing protein known as AHNAK [110,111,112]. Additionally, channel-activated increases in mitochondrial membrane potential are abolished when cells are exposed to latrunculin A, which disrupts actin assembly [104]. These findings have led to the proposal that a conformational change during activation of the L-type Ca^2+^ channel results in movement of the β_2_ subunit, which is transmitted to the mitochondria via the actin cytoskeleton. In this manner, activation of the channel can lead to actin-mediated increases in mitochondrial membrane potential.

**Figure 4:**
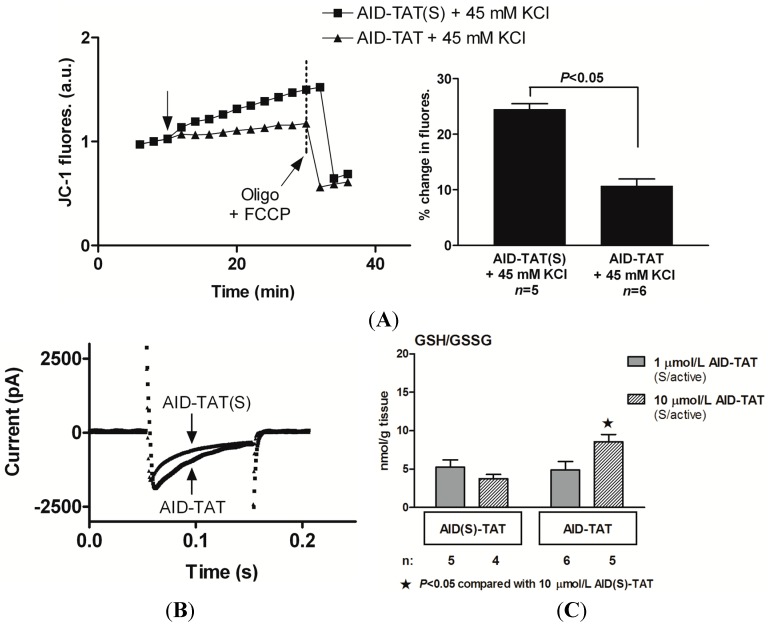
Targeted peptide delivery disrupts cross-talk between the L-type Ca^2+^ channel and the mitochondria and reduces ischemia-reperfusion injury. (**A**) Exposure to AID–TAT attenuates the increase in mitochondrial membrane potential (*Ψ*_m_) associated with activation of the channel. *Ψ*_m_ assessed as changes in JC-1 fluorescence recorded from two representative guinea pig myocytes before and after exposure to either 1 µM scrambled AID–TAT (AID–TAT(S)) or 1 µM AID–TAT followed by 45 mM KCl to activate the L-type Ca^2+^ channel. Arrow indicates where KCl was added. Mean ± SEM of changes in JC-1 fluorescence for myocytes exposed to AID–TAT(S) and AID–TAT shown on the right; (**B**) L-type Ca^2+^ channel current traces activated by voltage step to +10 mV from a holding potential of −30 mV from representative myocytes that were pre-incubated with either AID–TAT(S) or AID–TAT; and (**C**) GSH/GSSG ratio of guinea pig hearts treated with 1 or 10 µM scrambled AID–TAT (AID(S)–TAT) or active AID–TAT within 5 min after commencement of reperfusion following 30 min of no-flow ischemia. Panels A and B reproduced with permission from [104] and panel C reproduced with permission from [114].

To assess whether specific pharmacological interventions might be equally effective at disrupting communication between the channel and mitochondria, we applied a peptide derived against the AID of the channel using a transactivator of transcription (TAT) sequence to facilitate membrane transfer (AID–TAT peptide) [104]. The peptide immobilises the β_2_ subunit [113] and induces uncoupling from the actin cytoskeleton, allowing for normal calcium conductance to be maintained while altering mitochondrial and metabolic responses [104]. Application of the AID–TAT peptide to guinea pig ventricular myocytes effectively attenuates the increase in metabolic activity after activation of the channel. This includes the increase in oxidation of flavoprotein (flavin adenine dinucleotide) that is measured as autofluorescence and mitochondrial membrane potential upon activation of the channel [104] (Figure 4a). Using the whole-cell patch clamp technique we confirmed that exposure to AID–TAT modified the biophysical properties of the channel, such that it exhibited a delayed rate of inactivation of current as would be expected from binding of the peptide to the AID region of the α_1C_ subunit [104] (Figure 4b).

We additionally reported the therapeutic benefits of this construct, and demonstrated a dose-dependent alleviation of oxidative stress following coronary injection of the AID–TAT peptide in guinea pig hearts. Exposure to 10 µM of active peptide following I/R injury resulted in an increase in GSH to GSSG ratio [114] (Figure 4c), whereas a lower concentration of the peptide (1 µM) was insufficient to alter the GSH to GSSG ratio but still effective in reducing infarct size and CK and LDH levels. Application of the AID–TAT peptide was also found to improve contractility following oxidative stress. In untreated hearts, I/R injury caused a reduced left ventricular developed pressure which failed to improve by 30 min post-ischemia. Application of 10 µM AID–TAT peptide significantly improved developed pressure in reperfused hearts at 20–30 min post-reperfusion [114].

### 4.3. Targeted Peptide Delivery Decreases Infarct Size and Restores Contractility in Vivo

We have demonstrated that the L-type Ca^2+^ channel regulates mitochondrial function via a calcium-dependent mechanism. Calcium influx via the channel increases NADH production, superoxide production and metabolic activity [104]. L-type Ca^2+^ channel activation also induces an increase in mitochondrial membrane potential and metabolic activity, and this is mediated via the actin cytoskeleton. [104,115]. Given that increased production of ROS is thought to be a primary contributor to ischemia-reperfusion injury [49,102], we predicted that attenuation of ischemia-reperfusion injury using the AID–TAT peptide would result from an effect on mitochondrial function.

We examined the effect of application of AID–TAT peptide after ischemia-reperfusion in rats [114]. In these experiments the AID–TAT peptide was injected into the left ventricle after reperfusion of ischemic hearts. Following a recovery period of up to 12 weeks, infarct size, heart weight and left ventricular function were assessed. Both the low and high doses of the AID–TAT peptide were sufficient to reduce infarct size, and this was evident as early as 6 weeks post-infarction. The high dose of AID–TAT was also sufficient to prevent development of hypertrophy associated with ischemia-reperfusion injury, as well as restore left ventricular function.

These *in vivo* findings in combination with our *ex vivo* and *in vitro* data suggest that 10 µM AID–TAT is an effective therapeutic dose to reduce *I*/*R* injury. We propose that the effects are mediated by disruption of the functional communication that exists between the L-type Ca^2+^ channel and the mitochondria via cytoskeletal proteins, preventing increases in mitochondrial function that are induced by increased activation of the channel. This ultimately translates into reduced injury pathology when assessed in the short-term (reduced CK and LDH, increased GSH:GSSG) and in the long-term improved contractility [114]. Importantly, a 10 µM dose would still allow for calcium influx to be maintained under these conditions, enabling appropriate excitation-contraction to be sustained.

Prior clinical use of calcium modulating drugs have largely been spurred on by early animal studies demonstrating that blockade of the L-type Ca^2+^ channel using antagonists such as nisoldipine [116,117,118] and diltiazem [119,120] or by blocking with MgSO_4_ [121,122] limits myocardial damage following ischemia and reperfusion. Despite these positive preclinical findings, subsequent clinical trials using these blockers have produced mostly disappointing results [123,124,125]. This may be in part due to the substantial negative inotropic effects of these agents, a serious drawback in scenarios in which contractility may already be impaired following an ischemic insult. Therapeutic delivery of agents that allow for appropriate excitation-contraction to be maintained following reperfusion (such as the AID–TAT peptide) may overcome this shortcoming. Indeed, mibefradil (Ro 40-5967) is one such agent that does not evoke the substantial negative inotropic effects of other classical L-type Ca^2+^ channel blockers and was found to exhibit positive outcomes in experimental animals [126,127] as well as in a series of phase I, II and III clinical trials [128]. The agent was subsequently removed from the market as a result of adverse drug interactions, but provided convincing evidence that restoration of contractility post-ischemia is critical to reduce morbidity and mortality. Use of L-type Ca^2+^ channel modifying peptides, such as AID–TAT, therefore warrants further investigation to assess their efficacy in limiting myocardial damage following ischemia-reperfusion.

## 5. Concluding Remarks

Calcium influx via the L-type calcium channel is the predominant means of calcium entry into cardiomyocytes and provides the trigger for appropriate excitation contraction in the heart. Our data has verified that there is cross-talk between calcium flux via the L-type calcium channel and ROS produced by the mitochondria, such that increased intracellular calcium induces increased ROS production. In parallel, increased mitochondrial ROS leads to glutathionylation of the L-type calcium channel, resulting in a persistent increase in channel open probability. This constitutive channel activity leads to further increases in mitochondrial function, and thereby amplifies the cross-talk between ROS and the L-type calcium channel. We have good evidence to suggest that ROS-induced effects on channel function are likely to contribute to the development of pathology *in vivo*, and indeed preliminary experiments indicate that use of targeted peptide therapies that disrupt cross-talk between the channel and the mitochondria are capable of limiting reperfusion injury and restoring normal mitochondrial function.
